# Classification of Alzheimer’s Disease Based on Abnormal Hippocampal Functional Connectivity and Machine Learning

**DOI:** 10.3389/fnagi.2022.754334

**Published:** 2022-02-22

**Authors:** Qixiao Zhu, Yonghui Wang, Chuanjun Zhuo, Qunxing Xu, Yuan Yao, Zhuyun Liu, Yi Li, Zhao Sun, Jian Wang, Ming Lv, Qiang Wu, Dawei Wang

**Affiliations:** ^1^School of Information Science and Engineering, Shandong University, Qingdao, China; ^2^Department of Physical Medicine and Rehabilitation, Qilu Hospital of Shandong University, Jinan, China; ^3^Key Laboratory of Real Time Brain Circuits Tracing (RTBNP_Lab), Tianjin Fourth Center Hospital, Tianjin Fourth Hospital Affiliated to Nankai University, Tianjin, China; ^4^Department of Psychiatry, Tianjin Medical University, Tianjin, China; ^5^Department of Health Management Center, Qilu Hospital of Shandong University, Jinan, China; ^6^Department of Radiology, Qilu Hospital of Shandong University, Jinan, China; ^7^Department of Radiology, The Second People’s Hospital of Rizhao City, Rizhao, China; ^8^Department of Neurology, Qilu Hospital of Shangdong University, Jinan, China; ^9^Shandong Chenze AI Research Institute Co. Ltd., Jinan, China; ^10^Shandong Key Laboratory of Brain Function Remodeling, Department of Neurosurgery, Qilu Hospital of Shandong University, Jinan, China; ^11^Department of Clinical Epidemiology, Qilu Hospital of Shandong University, Jinan, China; ^12^Department of Epidemiology and Health Statistics, School of Public Health, Shandong University, Jinan, China; ^13^Institute of Brain and Brain-Inspired Science, Shandong University, Jinan, China

**Keywords:** Alzheimer’s disease, hippocampus, functional connectivity, classification, SVM

## Abstract

**Objective:**

Alzheimer’s disease (AD) is a neurodegenerative disease characterized by progressive deterioration of memory and cognition. Mild cognitive impairment (MCI) has been implicated as a prodromal phase of AD. Although abnormal functional connectivity (FC) has been demonstrated in AD and MCI, the clinical differentiation of AD, MCI, and normal aging remains difficult, and the distinction between MCI and normal aging is especially problematic. We hypothesized that FC between the hippocampus and other brain structures is altered in AD and MCI, and that measurement of abnormal FC could have diagnostic utility for the classification of different AD stages.

**Methods:**

Elderly adults aged 60–85 years were assigned to AD, MCI, or normal control (NC) groups based on clinical criteria. Functional magnetic resonance scanning was completed by 119 subjects. Five dimension reduction/classification methods were applied, using hippocampus-derived FC strengths as input features. Classification performance of the five dimensionality reduction methods was compared between AD, MCI, and NC groups.

**Results:**

FCs between the hippocampus and left insula, left thalamus, cerebellum, right lingual gyrus, posterior cingulate cortex, and precuneus were significantly reduced in AD and MCI. Support vector machine learning coupled with sparse principal component analysis demonstrated the best discriminative performance, yielding classification accuracies of 82.02% (AD vs. NC), 81.33% (MCI vs. NC), and 81.08% (AD vs. MCI).

**Conclusion:**

Hippocampus-seed-based FCs were significantly different between AD, MCI, and NC groups. FC assessment combined with widely used machine learning methods can improve AD differential diagnosis, and may be especially useful to distinguish MCI from normal aging.

## Introduction

Alzheimer’s disease (AD) is a progressive and irreversible neurodegenerative disease that leads to cognitive and physiologic dysfunction. The predicted 2050 prevalence of AD is estimated at 135.46 million patients (Fan et al., 2019). The histopathology of AD includes neuronal and synaptic degeneration, as well as senile plaque formation featuring extracellular deposits of β-amyloid protein (Glenner and Wong, 1984; Braak and Braak, 1991b,^1998^) and intracellular neurofibrillary tangles comprised of hyperphosphorylated tau protein (Bancher et al., 1989; Braak and Braak, 1995). However, AD is idiopathic, and current therapies only alleviate symptoms or delay progression. Investigating the pathophysiology of AD is essential to elucidate its potential etiologies.

Brain functional connectivity (FC) is a descriptive measure of spatiotemporal correlations between distinct regions of the cerebral cortex (Friston et al., 1993; Wang et al., 2006) that can yield new insights into the relationship between brain functional deficits and underlying structural disruptions (Kaiser, 2011; Wee et al., 2011). Multiple lines of evidence suggest that AD and several psychiatric diseases are related to disruption or enhancement of FC (Bullmore and Sporns, 2009; Qiao et al., 2017). In turn, the efficacy of cognitive behavioral therapy for manic depressive disorder and post-traumatic stress disorder may result from strengthened FC between cortical centers of cognitive control and the amygdala, potentially enhancing top-down control of dysregulated affective processes (Shou et al., 2017). However, most studies have focused narrowly on brain regions with abnormal connectivity, and have not further extracted the characteristics of these regional abnormalities to facilitate differential diagnosis. Clinically relevant information encoded in abnormal connectivity could be used to improve clinical diagnosis and disease classification.

Machine learning has been widely used in medical image processing due to its sensitive identification of patterns within large data sets. Numerous algorithms have been proposed to extract features from magnetic resonance and computed tomographic (CT) images to classify stages of AD. However, meaningful intermediate functional and structural information of the brain is unavailable (Cao et al., 2017, 2018). Novel machine learning algorithms provide new approaches to analyze differences in FC; these interdisciplinary research tools may promote the further development of medical imaging. For example, by extracting 93 volumetric features, Zhang et al. (2011) adopted a linear support vector machine (SVM) to evaluate the classification of AD. However, most studies only use structural magnetic resonance imaging (MRI) results such as voxel-wise tissue probability (Klöppel et al., 2008), cortical thickness (Desikan et al., 2009), and hippocampal volumes (Gerardin et al., 2009) to classify AD or MCI.

Hippocampal function is essential to memory performance; impairments are associated with AD. The utility of hippocampal shape and volume measurements to support the diagnosis of AD has been evaluated in multiple MRI studies (Gerardin et al., 2009; Amoroso et al., 2018). Platero and Tobar (2016) proposed a fast multiple-atlas segmentation method to measure hippocampal volume for the discrimination of AD/MCI patients from elderly controls. However, volumetric analysis is limited to the evaluation of local changes, and may be confounded by a high degree of normal variation. In addition to structural MRI, another important modality for the diagnosis of AD or MCI is resting-state functional MRI (fMRI). To date, the vast majority of studies that combine MRI with machine learning to classify AD or MCI have used region of interest-based rather than voxel-wise analysis (Meier et al., 2012; Wang C. et al., 2019). Voxel-wise FC analysis (Bejr-Kasem et al., 2018; Bagarinao et al., 2020) using the hippocampus as a seed region could represent a more targeted approach to demonstrate relationships between the hippocampus and the whole brain, and could potentially facilitate the differential diagnosis of MCI and AD.

## Materials and Methods

### Study Design

Elderly adults aged 60–85 years were assigned to AD, MCI, or normal control (NC) groups based on clinical criteria (see below), and then underwent fMRI scanning. We first selected the hippocampus as the seed region and constructed FC maps in a voxel-wise manner. We then used five machine learning algorithms to reduce dimensionality using hippocampus-derived FC strengths as input features to classify patients with AD or MCI, and normal controls (NC), and compared the accuracy of the five algorithms. The study protocol was approved by the Medical Ethics Committee of Qilu Hospital of Shandong University.

### Participants

Written informed consent was obtained from all subjects or their families using an informed consent approved by the Medical Ethics Committee of Qilu Hospital of Shandong University. Inclusion criteria included (1) right-handedness; (2) age of 60–85 years; and (3) ability to perform neuropsychological testing and tolerate MR scanning.

Exclusion criteria included: (1) metabolic diseases, such as hypothyroidism and vitamin B12/folic acid deficiencies; (2) history of psychiatric disorders such as depression or schizophrenia; (3) history of neurologic diseases associated with cognitive deficits (e.g., Parkinson’s syndrome or epilepsy); (4) history of drug or alcohol abuse; (5) receipt of neurotropic medications; (6) contraindications to MRI; and (7) CT or MRI abnormalities consistent with brain infarction or hemorrhage. Enrollment was limited to Chinese Han subjects to avoid population stratification artifacts.

After enrollment, subjects were assigned to either the AD, MCI, or NC group. AD subjects were selected according to the following inclusion criteria: (1) diagnosis of probable AD according to the National Institute of Neurological and Communicative Disorders and Stroke and the Alzheimer’s Disease and Related Disorders Association criteria; and (2) Clinical Dementia Rating (CDR) of 1 or 2. Subjects were assigned to the MCI group as described previously (Petersen et al., 1999) based upon: (1) subjective memory complaints of at least 6 months duration; and (2) CDR of 0.5. Inclusion criteria for NCs included: (1) normal physical status; (2) CDR of 0; and (3) absence of subjective memory complaints. Demographic and clinical variables that were evaluated for all groups included gender, age, educational level, Mini-mental Status Examination (MMSE) score, and CDR.

### Data Acquisition

A Siemens verio 3.0 Tesla MR scanner (Erlangen, Germany) was used to generate MR images. Tight and comfortable foam padding was used to minimize head movement, and earplugs were provided to mask scanner noise. Resting-state fMRI data were obtained using Gradient-Echo Single-Shot Echo-Planar Imaging sequence (GRE-SS-EPI) with the following imaging parameters: repetition time (TR)/echo time (TE) 2000/30 ms; field of view (FOV) 220 mm × 220 mm; matrix 64 × 64; flip angle (FA) 90°; slice thickness = 3 mm; and slice gap 1 mm; 36 transversal slices; 180 volumes. Subjects were instructed to close their eyes, remain awake and as stationary as possible, and not to concentrate on particular thoughts for the duration of the fMRI scan. Sagittal 3D T1-weighted images were generated by magnetization-prepared rapid acquisition gradient echo sequence (TR/TE 2000/2.3 ms; inversion time 900 ms; FA 9°; matrix 256 × 256; slice thickness 1 mm, no gap; 192 slices).

### Data Preprocessing

Preprocessing of functional images was accomplished by using the Resting-State fMRI Data Analysis Toolkit plus V1.23 (RESTplus)^1^ based on MATLAB. Considering the magnetic field instability and the frequency of movement artifacts during the initial scanning period, the first 10 volumes were removed and the remaining 170 volumes were evaluated. Slice-timing correction was performed to ensure the consistency of slice acquisition times. The resulting fMRI data sequences were realigned to compensate for head movement. Because small head movements contaminate FC results (Power et al., 2012), scans with more than 3 mm maximum translation in x, y or z, or 3° of maximum rotation about three axes were excluded. Framewise displacement (FD) was calculated to index volume-to-volume displacement in the head position. The FD was calculated from the derivatives of the rigid body realignment estimates used to realign fMRI data (Power et al., 2012). fMRI data sequences were then normalized by DARTEL using T1 image “New Segment” and smoothed with a 4-mm full-width half-maximum Gaussian kernel. Detrend was then performed to improve image quality, and to estimate nuisance covariates that included cerebrospinal fluid and white matter signals and six head motion parameters. Finally, band-pass filters were performed between 0.01 and 0.08 Hz.

### Functional Connectivity

As an important structure for memory function, the hippocampus may be especially relevant to the study of Alzheimer’s disease. Consequently, we selected the hippocampus as a seed region. However, because the neurophysiologies of the left and right the hippocampus may differ, we used both hippocampal aspects (from the Anatomical Automatic Labeling template) as seeds, and performed voxel-wise calculations of FC. The Pearson correlation coefficient between the time series of seeds and each voxel measures the consistency of their activities, which is defined as functional connection strength. Three-dimensional FC maps were generated for each seed. Fisher-z transformation was used to improve the normality of functional connectivity (Lowe et al., 1998).

### Statistical Analysis

Demographic and clinical characteristics were compared by the Chi-square and non-parametric Kruskal–Wallis tests. The latter was used to compare variables that are not subject to normal distributions. Statistical analyses were performed with Statistical Package for the Social Sciences (SPSS, Chicago, IL, United States, version 22.0) and significance threshold was set to *p* < 0.05.

Left and right hippocampus seed-based FC maps were subjected to statistical analysis. Analysis of covariance (ANCOVA) was used to differentiate FC values among the three groups. Meanwhile, the union of the significant regions of the one-sample *t*-test in the three groups was used as the mask of ANCOVA. Significant difference of statistical results was set at *p* < 0.05 (with a combined threshold of *p* < 0.05 and a minimum cluster size of 71 voxels), which was corrected by the AlphaSim program in RESTplus software. Brain regions with significant differences were extracted as masks. Two-sample post-hoc *t* tests of intra-mask FC maps were performed by Data Processing and Analysis for Brain Imaging^2^ between each pair of the three groups (AD vs. MCI, AD vs. NC, and MCI vs. NC). The intergroup significance level was set at *p* < 0.05 (with a combined threshold of *p* < 0.05 corrected by the AlphaSim program).

### Dimensionality Reduction

Machine learning methods have multiple applications in AD because they can learn data features quickly and efficiently, and can also fit the distributions of new data. Various machine learning algorithms have been applied to AD diagnosis. However, if a small number of four-dimensional fMRI images are directly classified using machine learning methods, performance will be poor and prone to over-fitting. We took the FC values of the significant difference regions as individual characteristics, supplemented by machine learning methods, to reduce over-fitting and realize the classification of small-size samples. Taking the regions with significant differences as masks, related subject FC values in each mask were extracted and spliced into a feature vector, which is the basis of using machine learning for classification. Feature vectors contain dozens or hundreds of functional connection values; consequently, the sample size of our subjects is relatively small compared to the number of features. This type of data can easily cause the Curse of Dimensionality problem. Dimensionality reduction can save computer memory requirements and prevent model over-fitting by eliminating redundant features and reducing computational complexity. Therefore, it is necessary to select features that are more useful for classification, and to map high-dimensional features to a low-dimensional space effectively. The following methods have good performance in dimensionality reduction: variance-threshold, mutual information, principal component analysis, and sparse principal component analysis.

#### Variance-Threshold

Features whose variance is less than the set value were removed by the variance threshold method. The variance calculation method is shown in equation (1). Small variance indicates that data are concentrated near a value; consequently, their contribution to classification is relatively small. Insufficient threshold indicates that only a small number of features are excluded, while excessive threshold indicates a tendency to lose important information. Consequently, the selection of an appropriate variance threshold is critical.


(1) $$\text{Var}\,\left(a\right)=\frac{1}{m}\,\sum_{i=1}^{m}{\left(a_{i}-\mu \right)}^{2}$$

#### Mutual Information

Mutual information is used to evaluate the correlation between independent and dependent variables. MI can measure the correlation between features and labels, to select features highly related to labels. The MI method is usually implemented by selecting a suitable threshold and defined as equation (2).


(2) $$\text{I}\,\left(X;Y\right)=\sum_{x\,\epsilon \,X}\sum_{y\,\epsilon \,Y}p\,\left(x,y\right)\,\log\frac{p\,\left(x,y\right)}{p\,\left(x\right)\,p\,\left(y\right)}$$

#### Principal Component Analysis

Principal component analysis (Jolliffe, 1988) is a widely used dimensionality reduction technique with a strict mathematical foundation. Data is transferred from the original *p*-dimensional coordinate system to a new coordinate system, with the largest variance taken as the first coordinate axis direction, because the maximum data variance yields the most valuable information. The second new coordinate axis selects the direction which is orthogonal to the first new coordinate axis and has the second largest variance. This process is repeated n times. This process can be realized by singular value decomposition of the feature matrix, as shown in equation (3). Let *X* be a *n×p* matrix, where *n* is the number of subjects and *p* is the number of features. Through the resulting coordinate system, most of the variance is contained in the first several coordinate axes, while the variance of the later axes is almost zero. Consequently, dimensionality may be reduced by selecting the first few axes and excluding the later axes for analysis.


(3) $$\text{X}=\text{UD}\,V^{T}$$

where, *T* indicates transpose, *U* indicates the principal components of unit length, and the columns of V are the corresponding loadings of the principal components. Most of the information will be contained in the first few principle components.

#### Sparse Principal Component Analysis

The SPCA algorithm reduces dimensionality and the size of explicitly used variables (Zou et al., 2006). PCA can be formulated as a regression-type optimization problem, thus the elastic net (Zou and Hastie, 2003) can be directly integrated into the regression criterion to produce sparse loadings (Zou et al., 2006). The optimization function of SPCA is expressed by equation (4), where *X* is the data matrix, and the second and third items are elastic net penalty terms. Whereas, the same λ is used for all *k* components, different λ_1,*j*_ are allowed to penalize the loadings of different principal components. Optimization can be solved by fixing α and β, and finally sparse principal components can be obtained.

Efficient algorithms to realize SPCA for both regular multivariate data (n>p) and gene expression arrays (*n*≪*p*) have been developed. Our data are directly applicable to the latter (Zou et al., 2006).


(4) $$\left(\hat{\alpha },\hat{\beta }\right)=\arg\,\min_{\alpha ,\beta }\sum_{i=1}^{n}{\left|X_{i}-\alpha \,\beta^{T}\,X_{i}\right|}^{2}+\lambda \,\sum_{j=1}^{k}{\left|\beta_{j}\right|}^{2}+\sum_{j=1}^{k}\lambda_{1,j}\,{\left|\beta_{j}\right|}_{1}$$

subject to α*^T^*α = I_*k*_.

We performed the above dimensionality reduction methods on the FC features to extract efficient patterns for classifications.

### Classification and Evaluation

Support vector machine with a radial basis function kernel was selected for binary classification. SVM optimizes models by introducing relaxation variables and adjusting penalty coefficients. For each pair of the three groups (AD vs. NC, AD vs. MCI, and MCI vs. NC), we extracted the functional connection values (left and right, respectively) from regions with significant differences in the two-sample post-hoc *t* test as mask (left and right, respectively), and then fused the left and right functional connection values to form a *np* feature matrix, in which *n* is the total number of subjects in the two compared groups, and *p* is the total number of features (FC values). After dimensionality reduction, feature matrices were classified by SVM.

**FIGURE 1:**
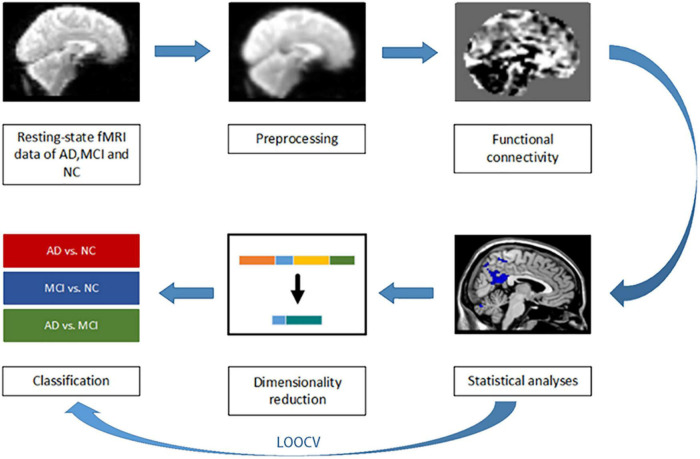
A preprocessing, feature extraction, and classification framework.

Leave-one-out cross validation (LOOCV) was used to evaluate the accuracy of the three binary classification tasks. One subject is used as the test set, and the remaining subjects are used as the training set for dimensionality reduction and classification experiments. Specifically in each iteration, the training set is performed dimensionality reduction and classification first, and then the testing set is used to test the effect of the training model. LOOCV performs *n* iterations (*n* is the number of individuals in the binary classification tasks) after the statistical analysis. Each iteration uses one subject to test the model, while the remaining subjects are used to train the model. This procedure was repeated until each subject was used as a test set once. The final classification accuracy shown in Table 1 was expressed as the percentage of subjects correctly predicted by these models. We perform let-one-out experiments before the statistical analysis to evaluate the experimental results consistency as shown in Figure 1. Left and right hippocampus seed-based FC maps have good repeatability and reproducibility in 10 iterations. The FC maps comparison of each iterations in detail were shown in Supplementary Material.

**TABLE 1:** Demographic and behavioral characteristics.

	AD	MCI	NC	*p* value
Number	44	30	45	
Male/Female	16/28	17/13	15/30	0.104
Age (Mean ± SD) years	67.43 ± 7.37	67.5 ± 7.28	65.0 ± 6.31	0.317
Education (Mean ± SD) years	9.93 ± 3.98	8.97 ± 3.88	10.49 ± 3.83	0.223
MMSE score (Mean ± SD)	19.27 ± 3.22	22.57 ± 2.45	28.07 ± 1.94	<0.001
CDR score (Mean ± SD)	1.20 ± 0.51	0.5	0	<0.001

*MMSE, Mini-Mental State Examination; CDR, Clinical Dementia Rating.*

## Results

### Participants

A total of 127 right-handed elderly Chinese adults were enrolled. Eight subjects (6 AD, 1 MCI, and 1 NC) were excluded from analyses due to excessive head motion during scanning. The remaining 119 subjects included 44, 30, and 45 in the AD, MCI, and NC groups, respectively. There were no significant intergroup differences in age, gender, or years of education. However, MMSE scores were significantly different between the three groups (Kruskal-Wallis test, *p* < 0.001) and between each pair of groups (Bonferroni corrected, *p* < 0.05/3) (Table 2).

**TABLE 2:** Recognition accuracy of dimensionality reduction methods.

	AD vs. NC (%)	MCI vs. NC (%)	AD vs. MCI (%)
SVM	78.65	76.00	77.03
VAR+SVM	78.65	76.00	78.38
MI+SVM	78.65	77.33	77.03
PCA+SVM	77.53	76.00	79.73
SPCA+SVM	**82.02**	**81.33**	**81.08**

*The bold values are the best results of our experiments.*

### Hippocampal Connectivity Analyses Within Normal Control, Mild Cognitive Impairment, and Alzheimer’s Disease Groups

**FIGURE 2:**
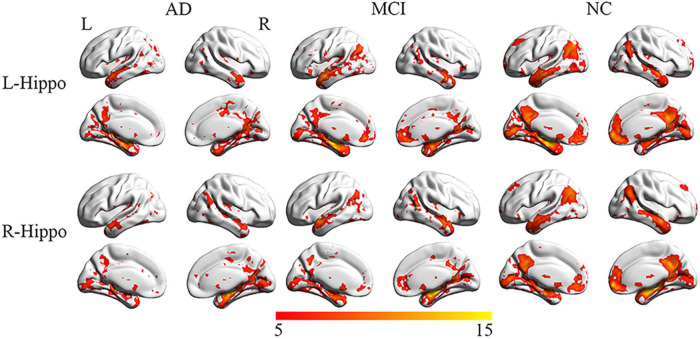
Representation of connectivity of the left and right hippocampus to multiple cortical regions in AD, MCI, and NC groups. Although connections to multiple regions are noted in AD and MCI, their number and extent are reduced compared to NC.

The NC group displayed significant connectivity between the left and right hippocampus and several brain regions that included the medial prefrontal cortex, posterior cingulate cortex (PCC), precuneus, inferior temporal cortex and inferior parietal cortex, and overlapped with regions of the default-mode network (Raichle et al., 2001). The number of regions with significant functional connections to the left and right hippocampus was substantially reduced in the AD and MCI groups compared to the NC group (Figure 2).

### Differences in Hippocampal Connectivity Among Alzheimer’s Disease, Mild Cognitive Impairment, and Normal Control Groups

#### Left Hippocampus as Seed

**FIGURE 3:**
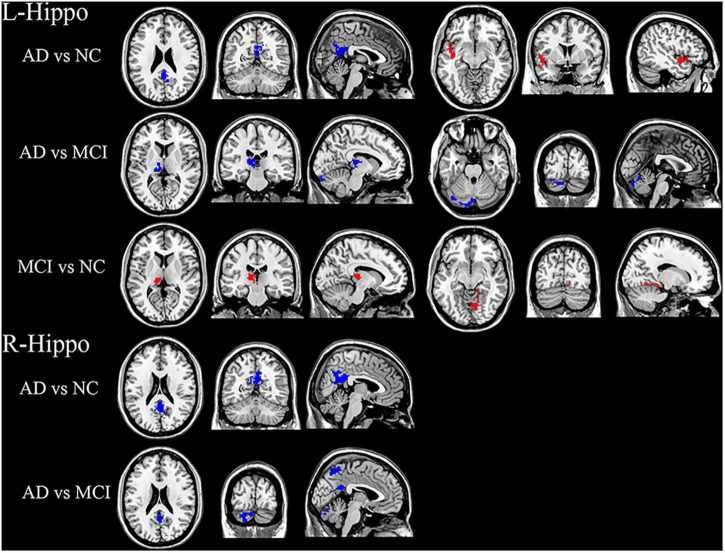
Significant hippocampal FC differences in AD, MCI, and NC groups. For left hippocampal FC: Decreased FC in the PCC, increased FC in the left insula in AD group compared with the NC group. Compared with the MCI group, the AD group showed decreased FC in the left thalamus and cerebellum. The MCI group showed increased FC in the right lingual gyrus and left thalamus compared with the NC group. For right hippocampal FC: FC in PCC was significantly decreased in the AD group compared with NC group. Significantly decreased FC in PCC, precuneus and cerebellum in AD group compared to the MCI group. Blue and red regions indicate decreased or increased FC, respectively.

Post-hoc *t* tests revealed that the AD group had decreased FC in the PCC and increased FC in the left insula compared with the NC group. Compared with the MCI group, the AD group showed significantly decreased FC in left thalamus and cerebellum. Compared with the NC group, the MCI group showed increased FC in the right lingual gyrus and left thalamus (Figure 3).

#### Right Hippocampus as Seed

The AD group showed significantly decreased FC in the PCC compared with the NC group. In addition, the AD group exhibited decreased FC in the PCC, precuneus, and cerebellum compared to the MCI group (Figure 3). No significant FC differences were demonstrated between the MCI and NC groups.

### Classification Accuracy

**FIGURE 4:**
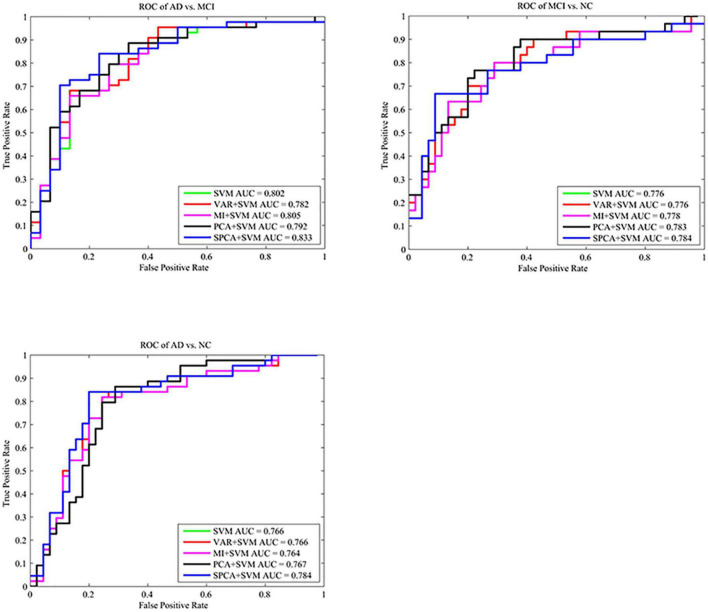
Comparison of the ROC curves based on SVM, VAR+SVM, MI+SVM, PCA+SVM, and SPCA+SVM. ROC curves and AUC show that SPCA combined with SVM has the best performance for the three tasks.

Sparse principal component analysis combined with SVM achieved the best classification performance, with an accuracy of 82.02% (AD vs. NC), 81.08% (AD vs. MCI), and 81.33% (MCI vs. NC). SPCA+SVM exhibited the largest area under the ROC curve and had the best classification performance as shown in Figure 4.

## Discussion

We analyzed FC maps of both the left and right hippocampus, which are of critical importance to memory function and early deficits in AD and MCI. Bilateral hippocampal-whole brain FC maps of AD, MCI, and normal elderly controls were subjected to dimensionality reduction and classification. The combination of SPCA and SVM yielded high classification accuracies, and may offer an accurate modality to facilitate the differential diagnosis of MCI and AD.

Abnormal FCs were located primarily in the PCC, precuneus, insula, lingual gyrus, thalamus, and cerebellum in the AD and MCI groups. The PCC and precuneus are core regions of the default-mode network initially described by Raichle et al. (2001) that has been closely linked with episodic memory (Daselaar et al., 2004; Greicius et al., 2004). Functional deficits of these regions are associated with cognitive deficits. For example, the PCC plays an important role in spatial orientation, self-appraisal, internal monitoring, and memory processing (Gusnard et al., 2001; Greicius et al., 2003). In addition, abnormal resting-state hippocampal-PCC FC has been reported in both AD and MCI (Wang et al., 2006; Sorg et al., 2007). Moreover, precuneal dysfunction plays a fundamental role in the memory impairment of early AD (Koch et al., 2018). Consistent with previous studies, our findings suggest that decreased FC in the PCC and precuneus might reflect impaired memory and cognition in AD and MCI.

More specifically, the MCI group showed increased FC in the left thalamus and right lingual gyrus compared with the NC group. Previous studies have indicated that the thalamus plays an important role in declarative memory and in modulating communication in all areas of the cerebral cortex (Van der Werf et al., 2003; de Rover et al., 2008). Amyloid deposits and neurofibrillary tangles have been found in the thalamus in MCI (Braak and Braak, 1991a), and significant thalamic gray matter loss has been reported in AD (Karas et al., 2004). Recently, Wang J. et al. (2019) found that FC was increased in the lingual gyrus in MCI, suggesting disease-related compensatory adaptations of brain networks, consistent with our results.

Notably, compared with the NC group, the AD group showed enhanced connectivity of the middle and posterior left insula. The insula regulates cerebral circulation and facilitates memory as well as emotional and sensory processes (Bonthius et al., 2005). AD is associated with insular gray matter loss (Guo et al., 2012), and disrupted connectivity (Xie et al., 2012; Liu et al., 2018). Our finding of increased FC of the left insula in AD is noteworthy because it might represent a compensatory adaptation to offset impaired memory and cognition. Lin et al. (2017) found higher fractional amplitude of low-frequency fluctuations in the insula and inferior frontal gyrus in a group at genetic risk of AD, suggesting that these regions play a critical role in the mitigation of neurodegeneration. Furthermore, neurofibrillary tangle density is significantly greater in the agranular cortex located in anterior insula than in the dysgranular and granular cortex of the middle and posterior insula of AD patients (Bonthius et al., 2005), which suggests increased vulnerability of the anterior insula. Thus, increased FC of the middle and posterior insula might represent a compensatory mechanism to re-establish normal brain function.

Computer-aided classification techniques that combine machine learning methods to MRI or PET have been applied for the diagnosis of AD or MCI (Ortiz et al., 2016; Lu et al., 2018). The most widely used classifier is SVM, which extracts information from MRI or PET images to build predictive classification models that facilitate clinical diagnosis (Cortes and Vapnik, 1995; Rathore et al., 2017). However, high-dimensionality and small-size training samples cause the Curse of Dimensionality and performance degradation. Therefore, reductions of the dimensionality of feature vectors are necessary. MI and VAR are usually applied to select efficient features, while PCA and SPCA can extract essential patterns from large data sets. Feature selection is used to identify representative features and to reduce the dimensionality of the feature space, and demonstrates good classification performance. The retention of an excessive number of features weakens the effect of dimensionality reduction, while the analysis of an insufficient number of features can result in the loss of important information. In the case of high-dimensional data, MI and VAR performance characteristics are limited. Feature extraction is used to transform high-dimensional feature vectors into low-dimensional feature vectors through a method of mapping or transformation. Traditional PCA is widely used in various applications, but the process of high-dimensional to low-dimensional mapping is actually a linear combination of original feature variables. The performance of PCA degrades when faced with high-dimensional data. SPCA is an extension of PCA, that has an extra sparse loading structure by using the Lasso (elastic net) (Zou and Hastie, 2003; Zou et al., 2006). The regularized sparse model facilitates the simplification of feature vectors and the alleviation of over-fitting. During the optimization procedure, Lasso penalty can search for uncorrelated directions and reduce discriminative projection vectors. In addition, the sparse constraint facilitates noise reduction in the function connection value and the extraction of robust features. Consequently, SPCA enables sparse results and facilitates the mapping of high to low dimensions. Our final experimental results also demonstrate the effectiveness and robustness of our proposed framework. Moreover, for data with size n × p (*n*≪*p*), setting λ to infinity as proposed in SPCA accelerates the calculation process. The highly accurate classification results generated by our algorithm suggest that abnormal FC between the hippocampus and whole brain is a clinically relevant and reliable diagnostic biomarker.

## Limitations

Our study had several limitations. First, the sample size in this study was relatively small. A much larger sample should be collected to improve the robustness and generalizability of the classification model. Second, multimodal features such as diffusion tensor imaging, cognitive scale scores, and cerebrospinal fluid tau protein levels should also be investigated, which may lead to higher classification accuracy. Finally, a comparison of our results with large benchmark datasets would be valuable. The implementation of a classification model based on public datasets should be considered for future research.

## Conclusion

We calculated FCs between the bilateral hippocampus and the whole brain in AD, MCI, and NC. Both the left and right hippocampus have significantly enhanced or attenuated FC to multiple important brain regions, primarily the PCC, left insula, left thalamus, and cerebellum. The abnormal FC values of each subject were extracted as discriminative patterns, which when combined with SPCA for dimensionality reduction and SVM for classification, facilitated the highly accurate differentiation of MCI from NC. Our results suggests that abnormal hippocampal connectivity may serve as a potential neuroimaging biomarker to expedite the early diagnosis of AD and MCI.

## Data Availability Statement

The original contributions presented in the study are included in the article/Supplementary Material, further inquiries can be directed to the corresponding authors.

## Ethics Statement

The studies involving human participants were reviewed and approved by Qilu Hospital of Shandong University. The patients/participants provided their written informed consent to participate in this study.

## Author Contributions

QW and DW conceived and designed the study. QZ, YW, and CZ performed testing and data collection and drafted the manuscript. QZ, QX, CZ, YY, ZL, YL, YW, ZS, JW, and ML performed the data analysis and interpretation. All authors contributed to the article and approved the submitted version.

## Conflict of Interest

ZS was employed by the company Shandong Chenze AI Research Institute Co. Ltd. The remaining authors declare that the research was conducted in the absence of any commercial or financial relationships that could be construed as a potential conflict of interest.

## Publisher’s Note

All claims expressed in this article are solely those of the authors and do not necessarily represent those of their affiliated organizations, or those of the publisher, the editors and the reviewers. Any product that may be evaluated in this article, or claim that may be made by its manufacturer, is not guaranteed or endorsed by the publisher.

## Footnotes

1
http://restfmri.net/forum/index.php

2
http://rfmri.org/dpabi
