# Supplementary material for: Classification of Alzheimer’s Disease Based on Abnormal Hippocampal Functional Connectivity and Machine Learning
Source: Front Aging Neurosci. 2022 Feb 22;14:754334. doi: 10.3389/fnagi.2022.754334 (PMC8902140; doi:10.3389/fnagi.2022.754334)
Supplement: Supplementary file 1 [file Data_Sheet_1.PDF]

Supplementary material

We performed leave-one-out for ten times and one subject is used as the test set, and the remaining subjects are used as the training set for dimensionality reduction and classification experiments. As shown in the following figures, we find the FC maps have good repeatability and reproducibility. In left-hippo FC, AD group had decreased FC in the PCC and increased FC in the left insula compared with the NC group. Compared with the MCI group, the AD group showed significantly decreased FC in left thalamus and cerebellum. Compared with the NC group, the MCI group showed increased FC in the right lingual gyrus and left thalamus. While in right-hippo FC, AD group showed significantly decreased FC in the PCC compared with the NC group. In addition, the AD group exhibited decreased FC in the PCC, precuneus, and cerebellum compared to the MCI group. No significant FC differences were found between the MCI and NC groups.

FC maps in 10 iterations

L-Hippo

AD-NC

AD group had decreased FC in the PCC and increased FC in the left insula compared with the NC group.

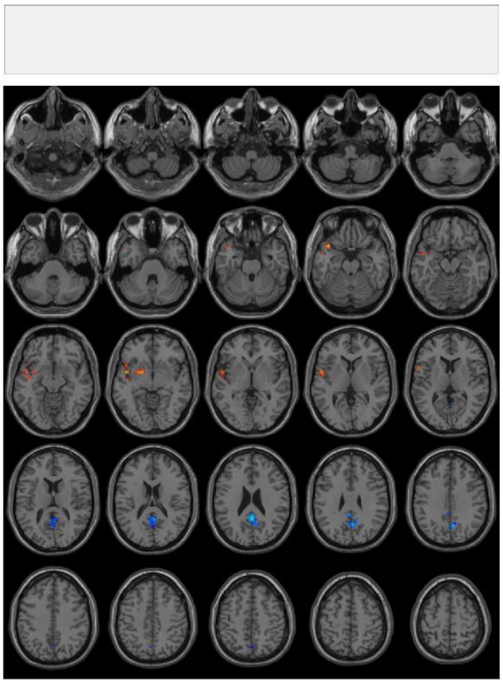

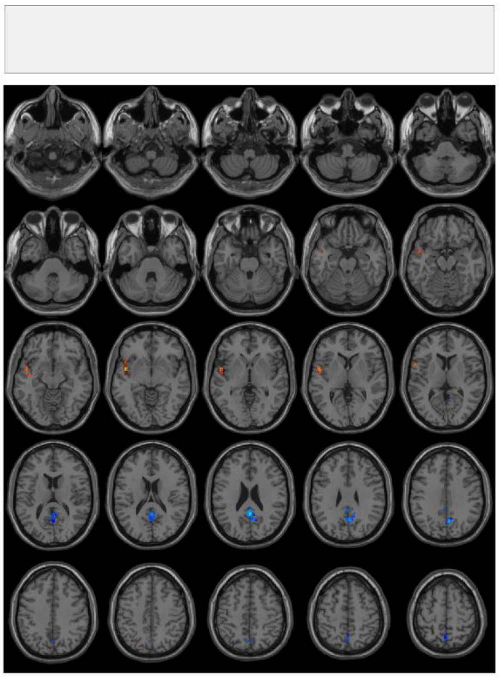

Test1 **Test2**

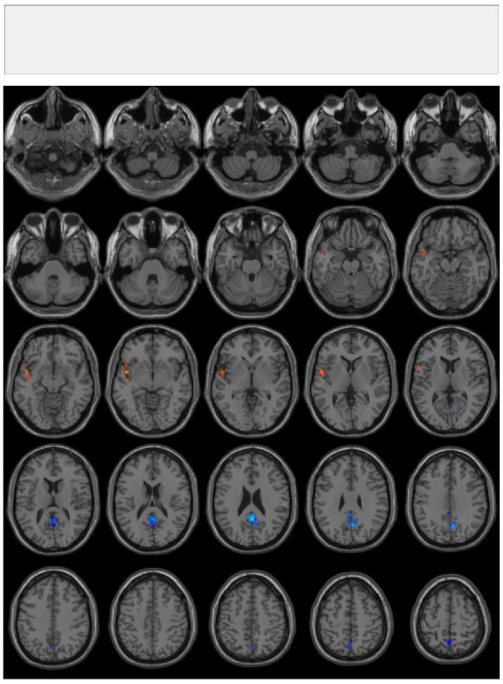

Test3

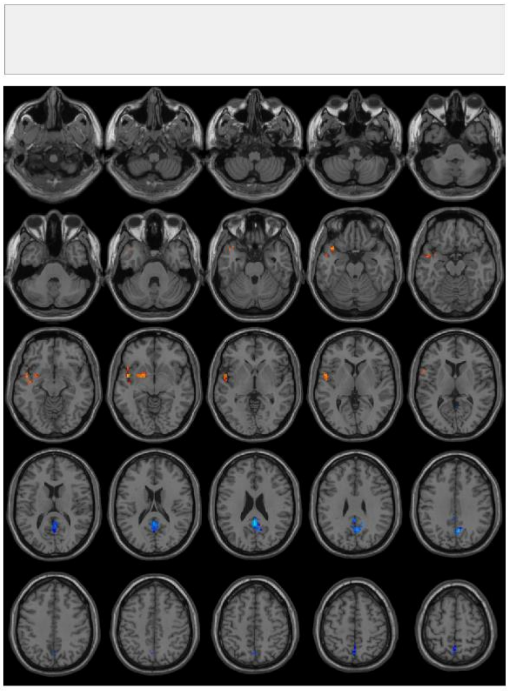

Test4

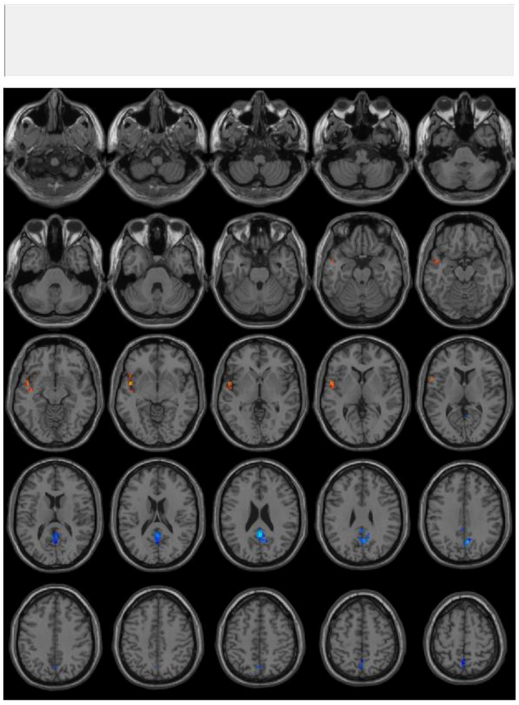

Test5

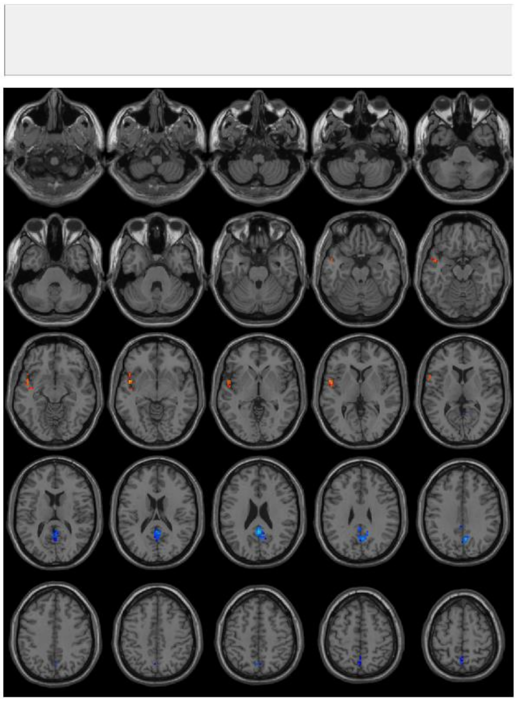

Test6

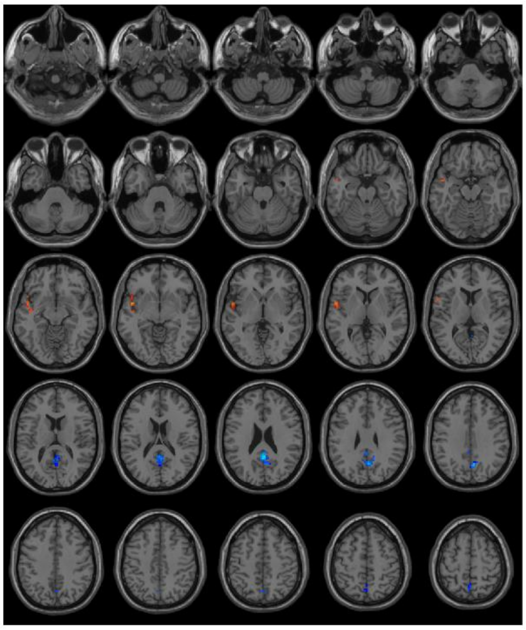

Test7

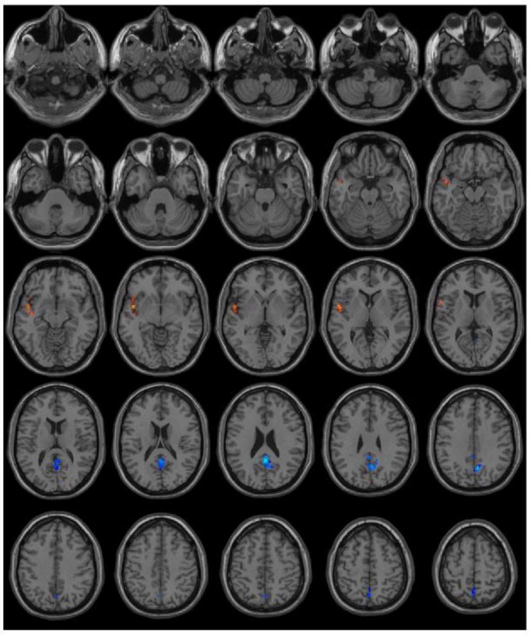

Test8

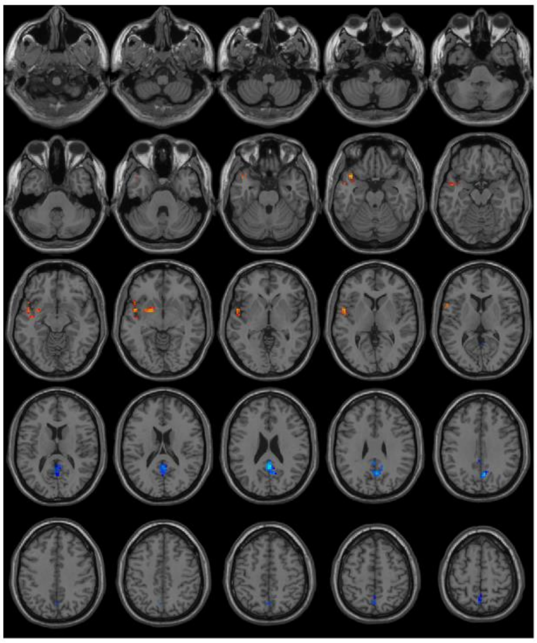

Test9

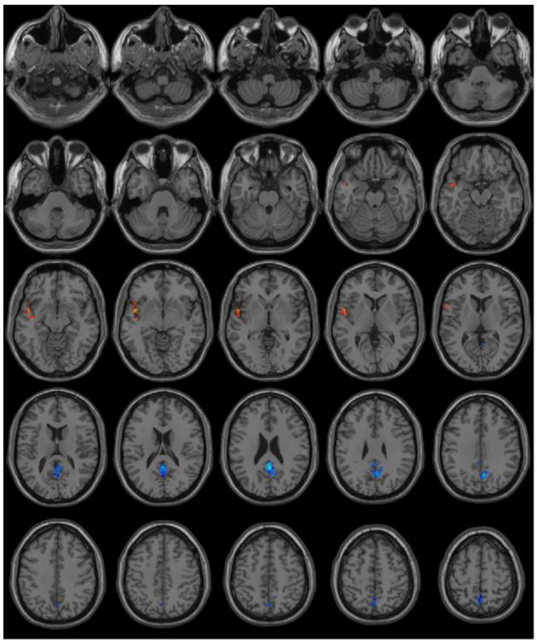

Test10

L-Hippo

AD-MCI：Compared with the MCI group, the AD group showed significantly decreased FC in left thalamus and cerebellum.

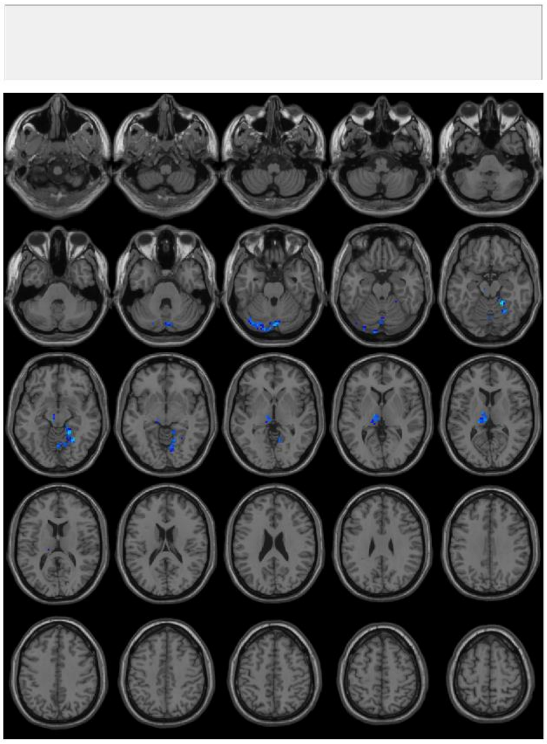

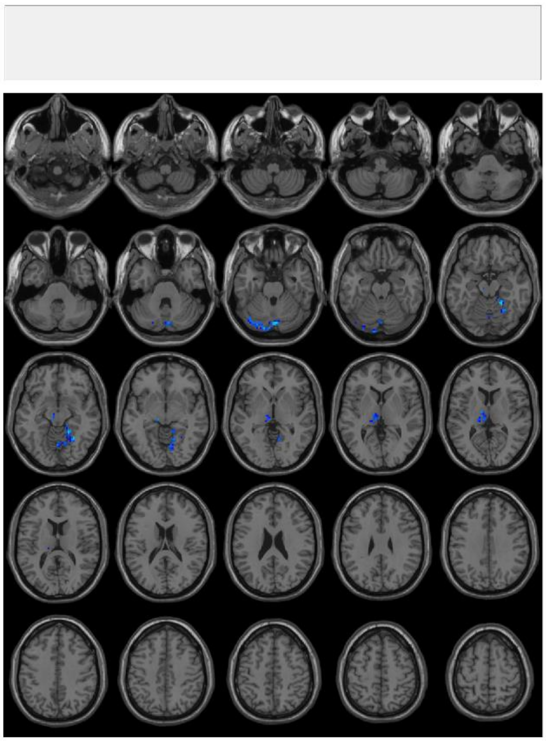

Test1

Test2

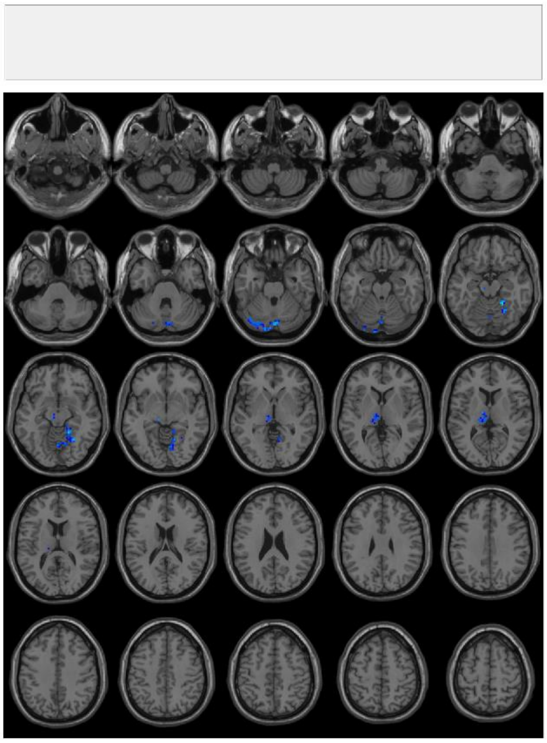

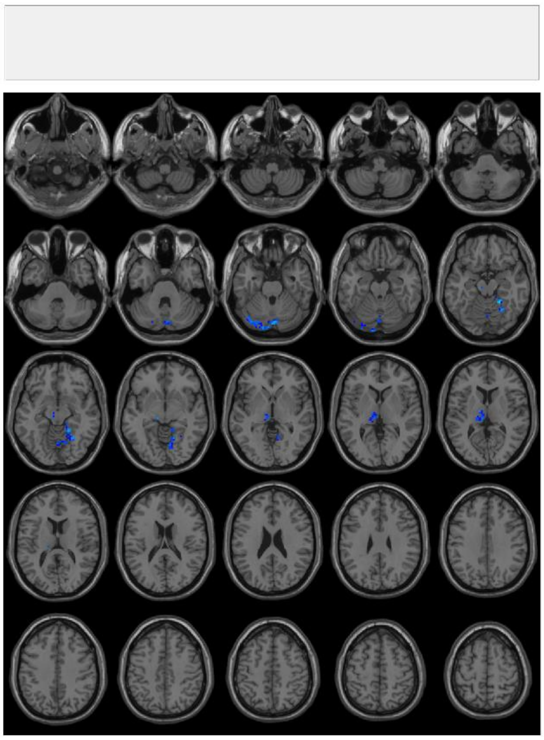

Test3

Test4

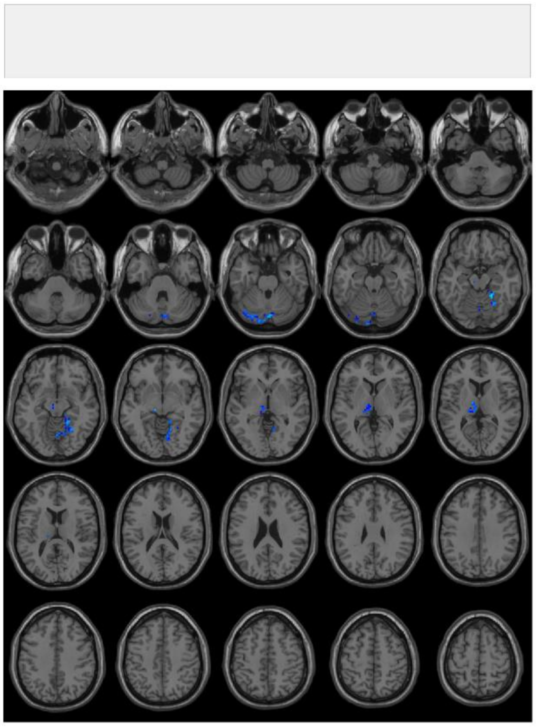

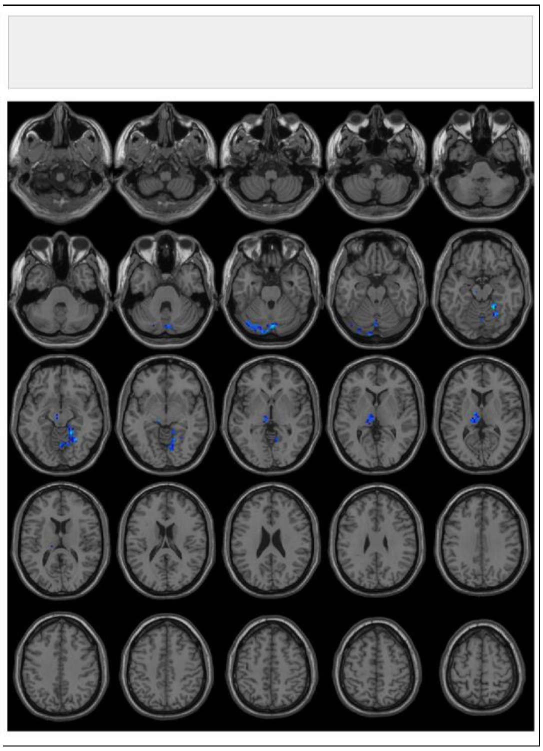

Test5

Test6

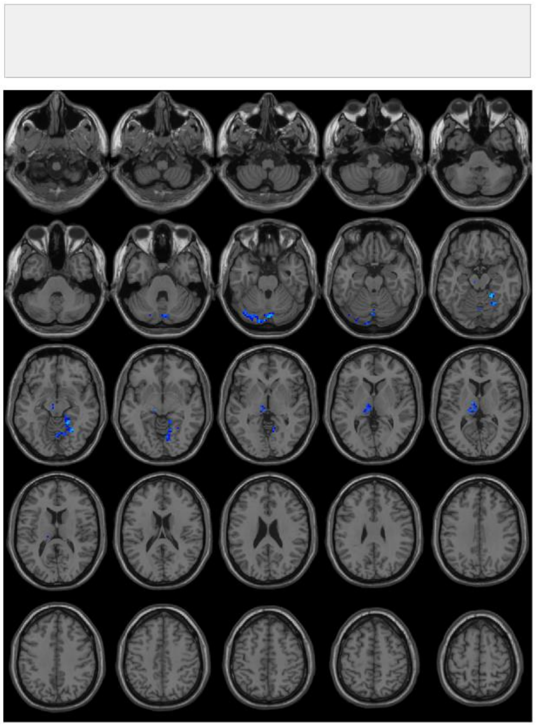

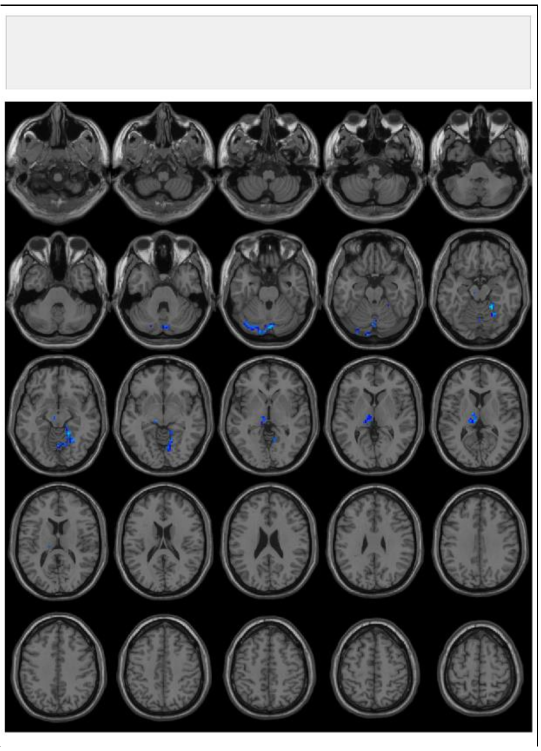

Test7

Test8

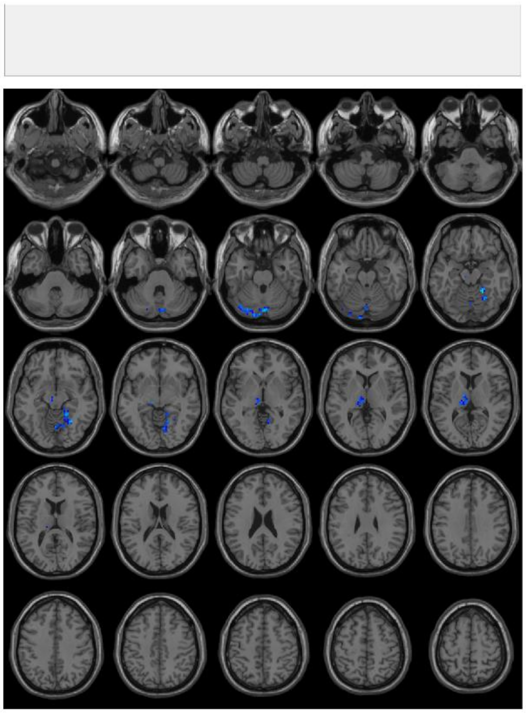

Test9

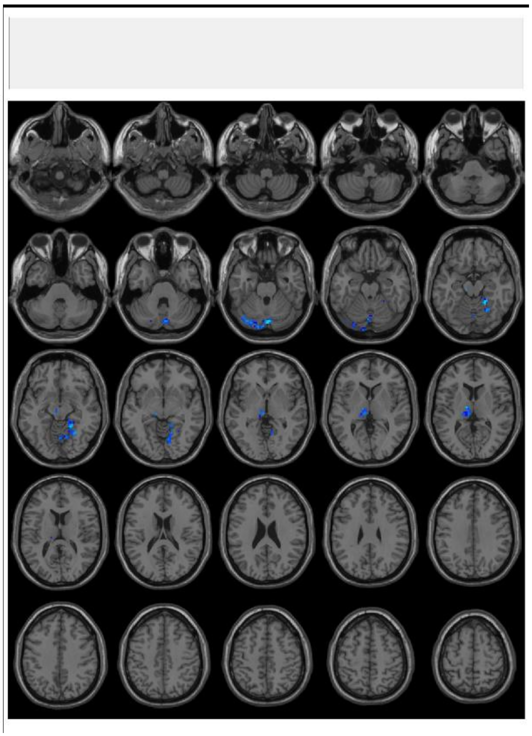

Test10

L-Hippo

MCI-NC：Compared with the NC group, the MCI group showed increased FC in the right lingual gyrus and left thalamus

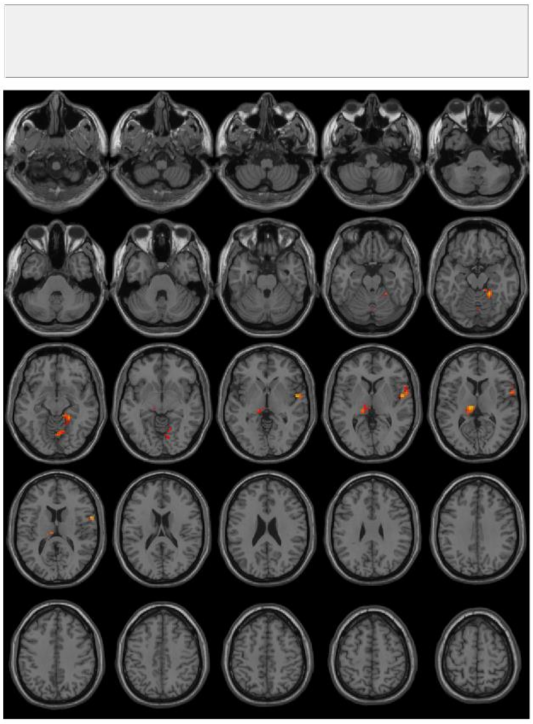

Test1

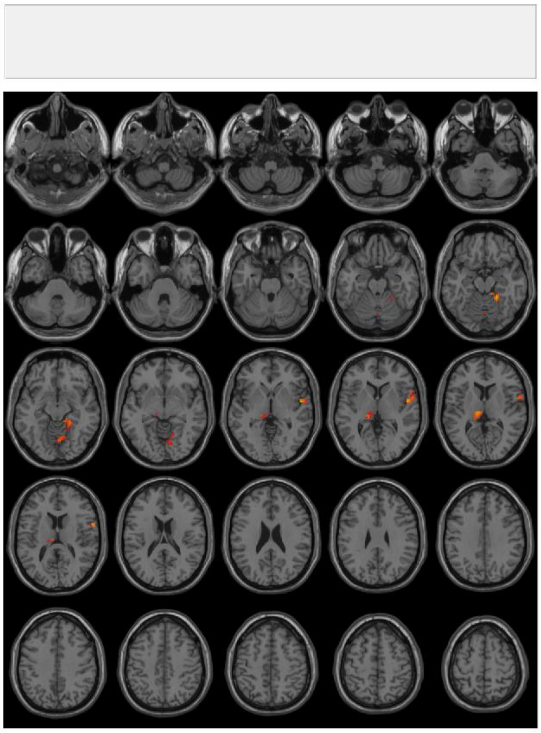

Test2

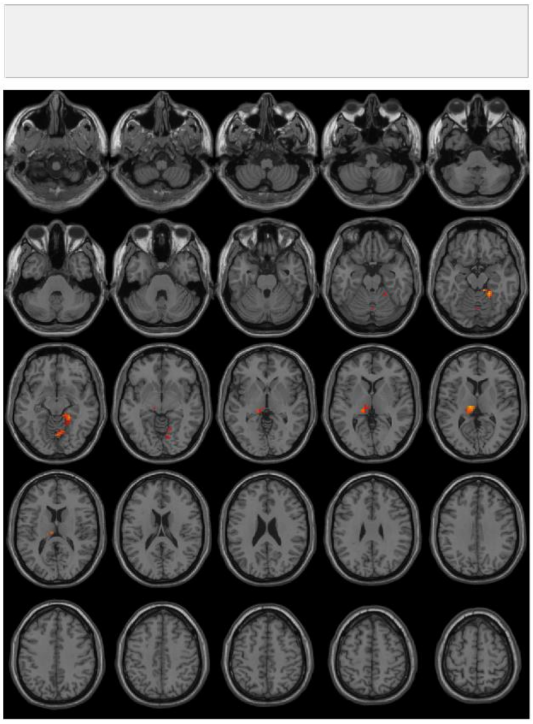

Test3

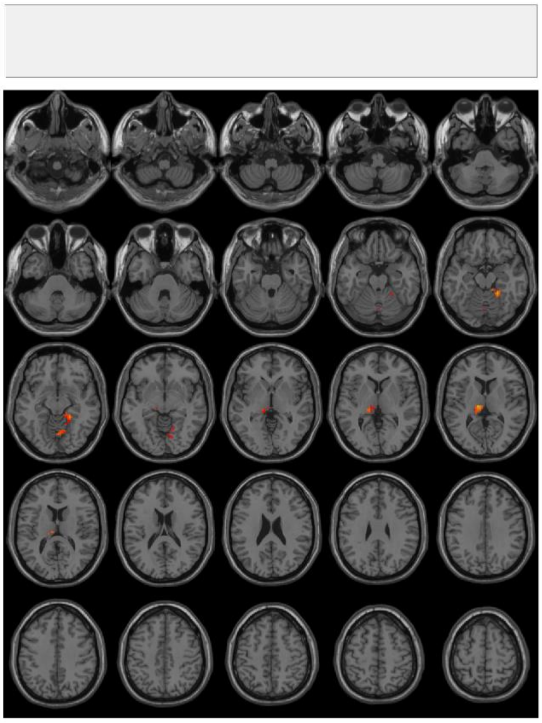

Test4

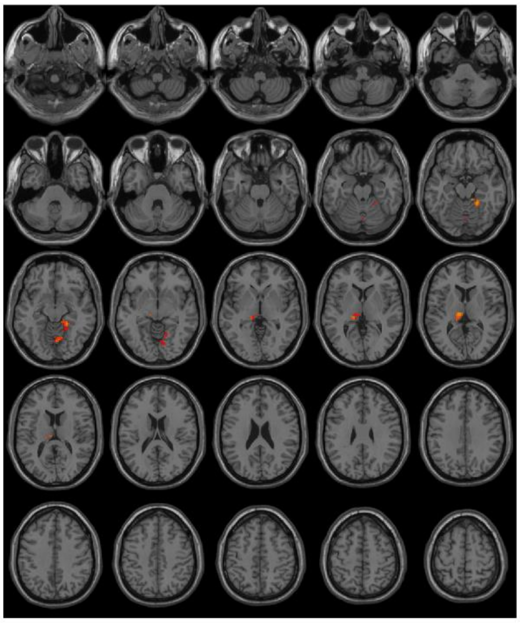

Test5

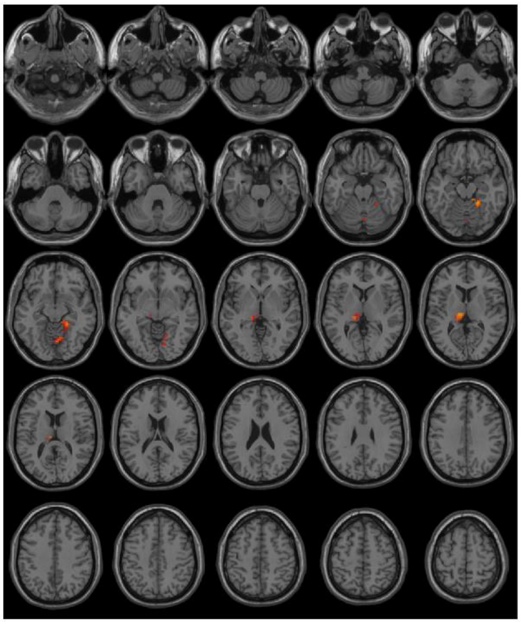

Test6

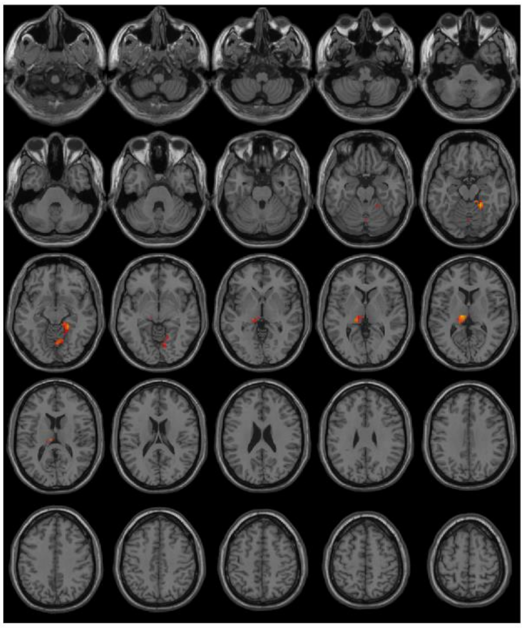

Test7

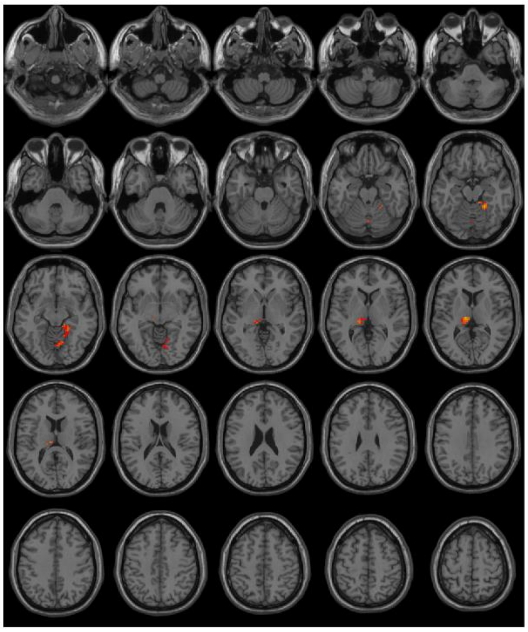

Test8

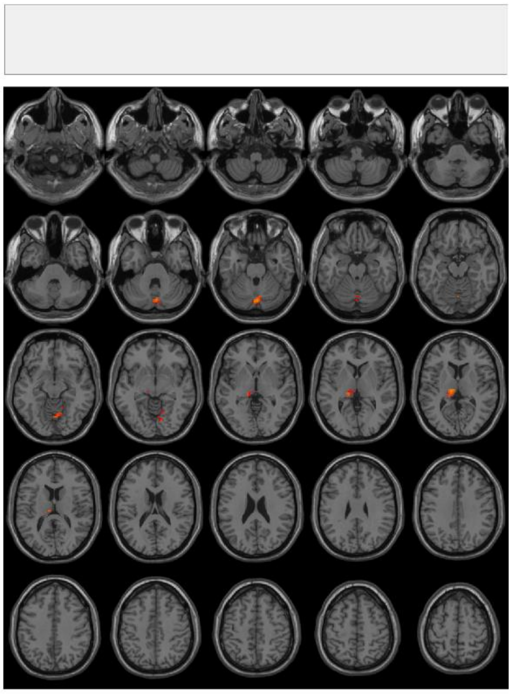

Test9

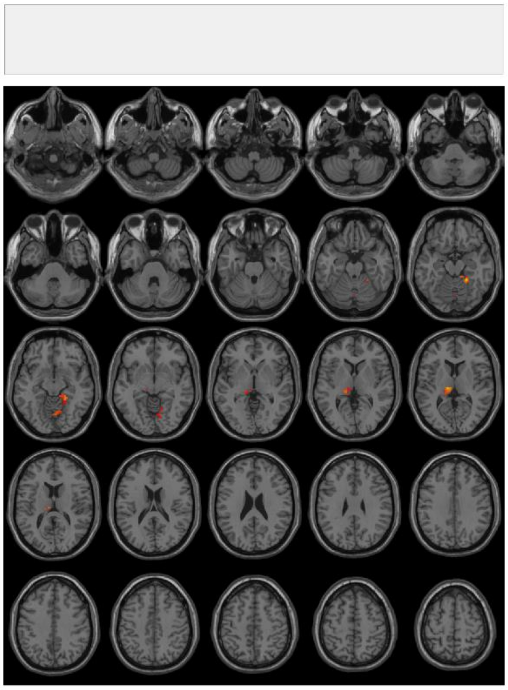

Test10

R-Hippo

AD-NC

AD group showed significantly decreased FC in the PCC compared with the NC group.

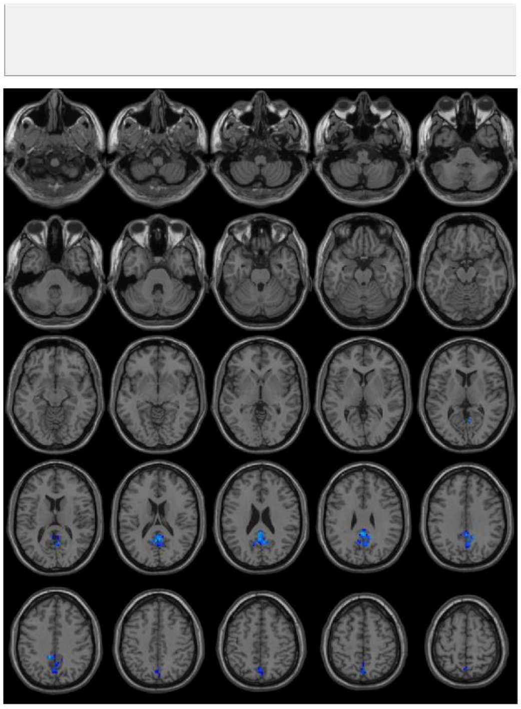

Test1

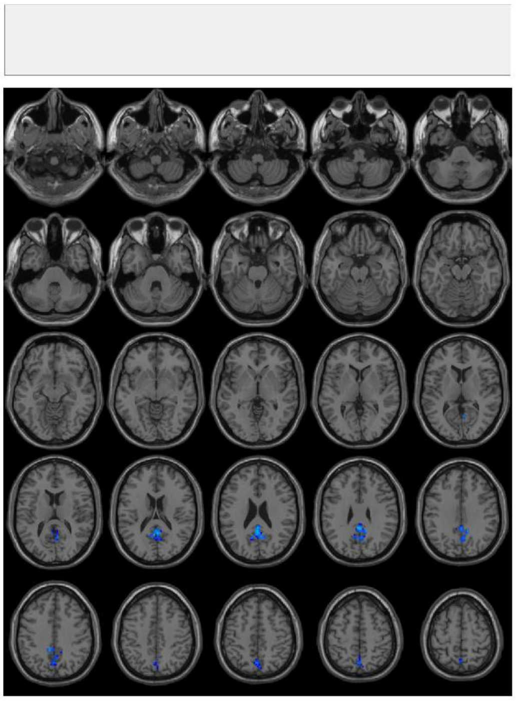

Test2

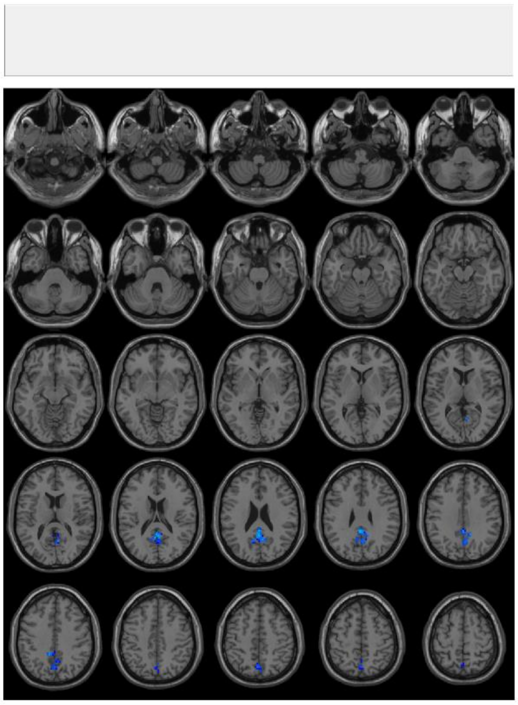

Test3

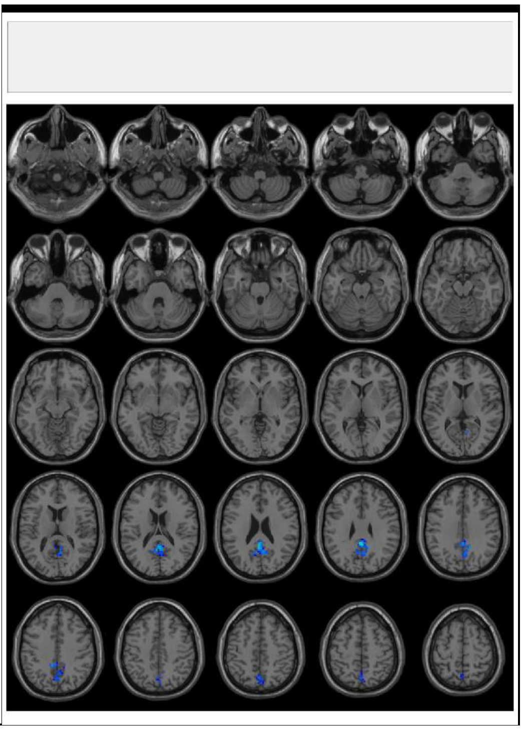

Test4

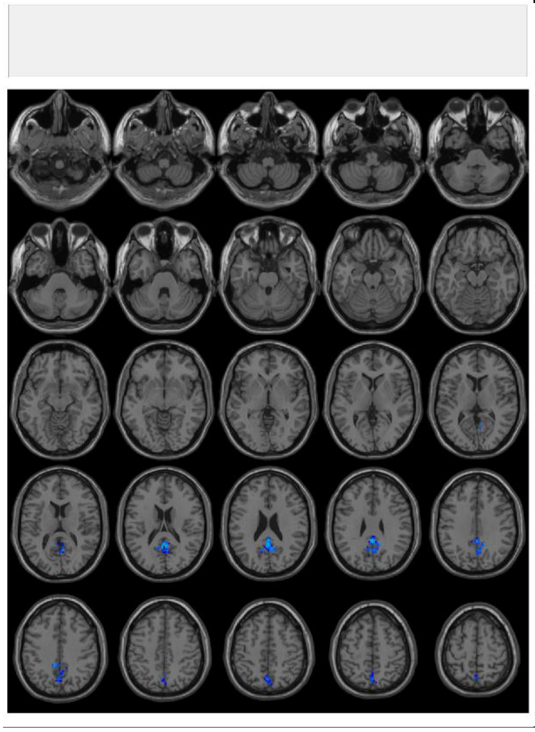

Test5

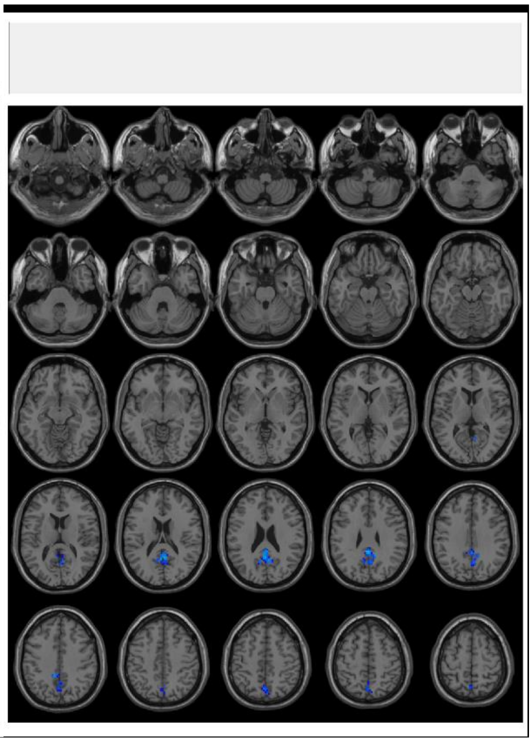

Test6

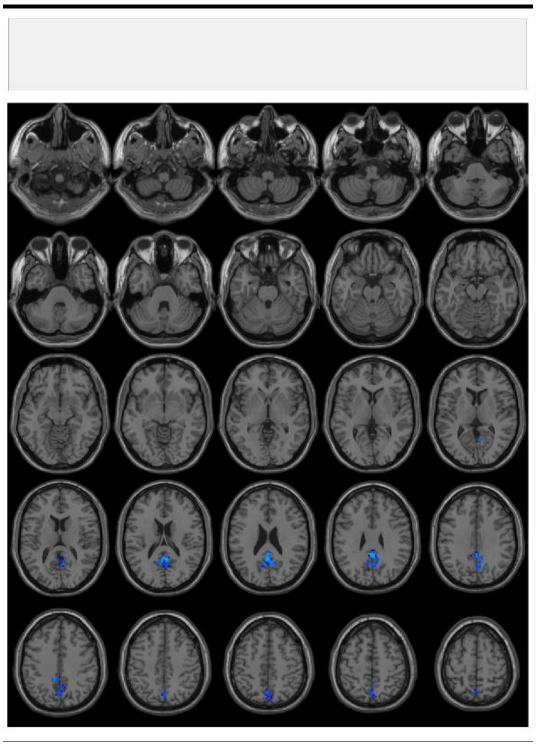

Test7

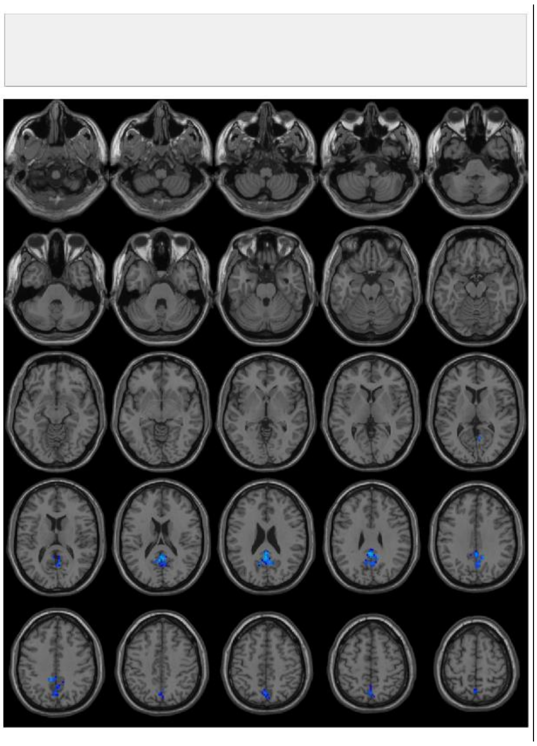

Test8

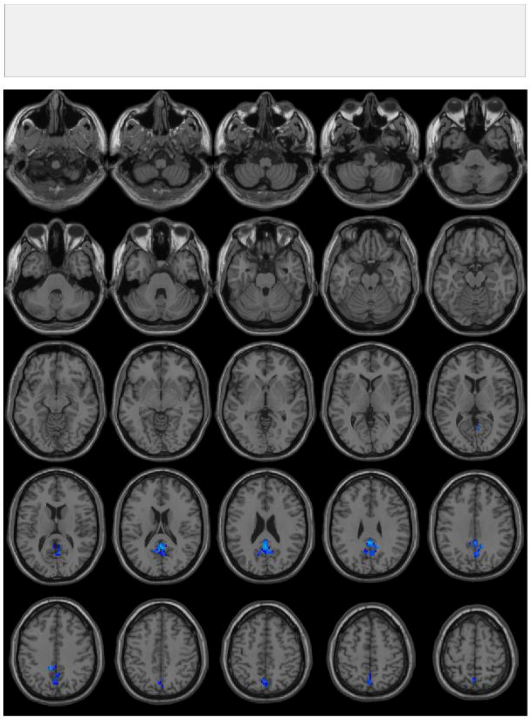

Test9

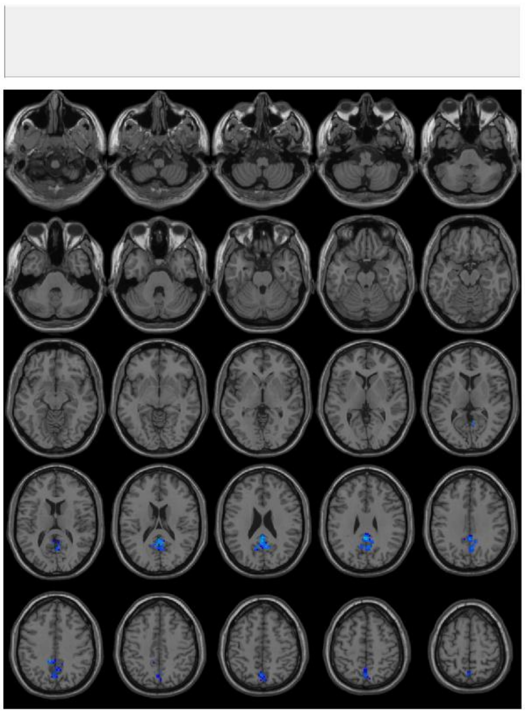

Test10

R-Hippo

AD-MCI

AD group exhibited decreased FC in the PCC, precuneus, and cerebellum compared to the MCI group.

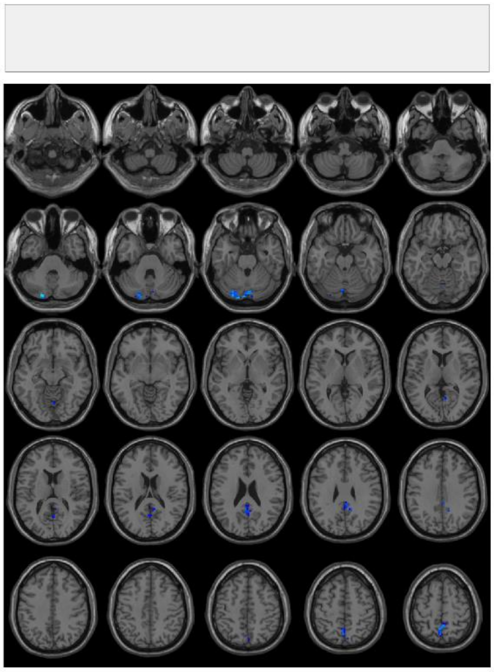

Test1

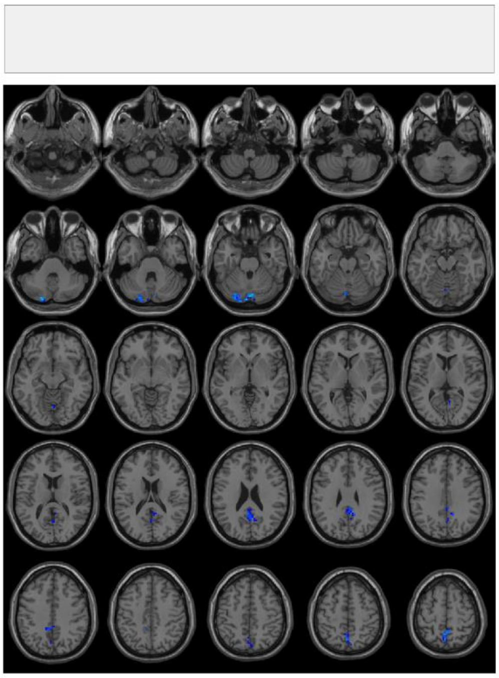

Test2

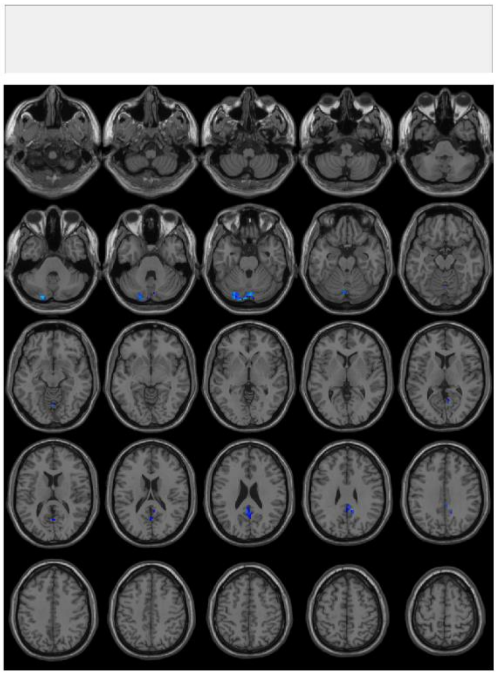

Test3

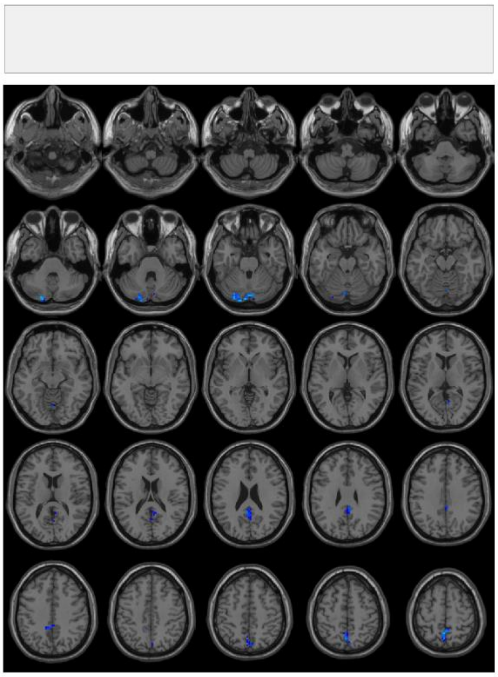

Test4

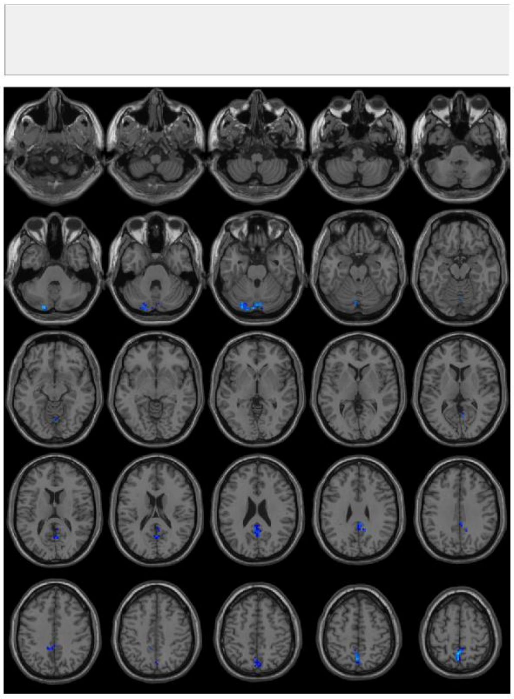

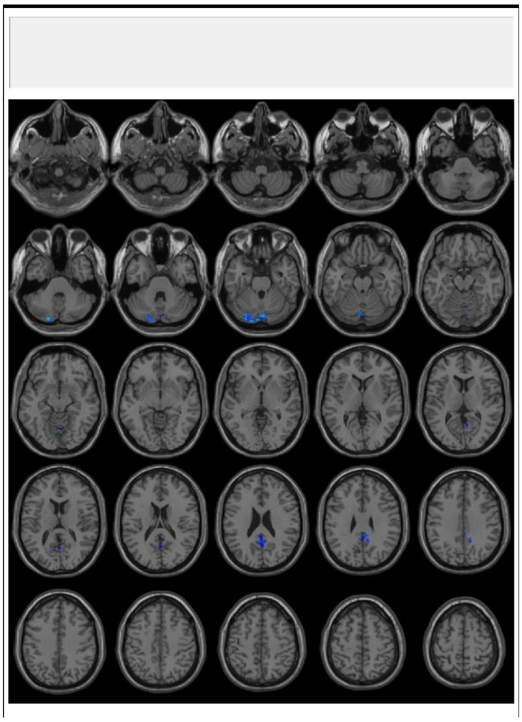

Test5

Test6

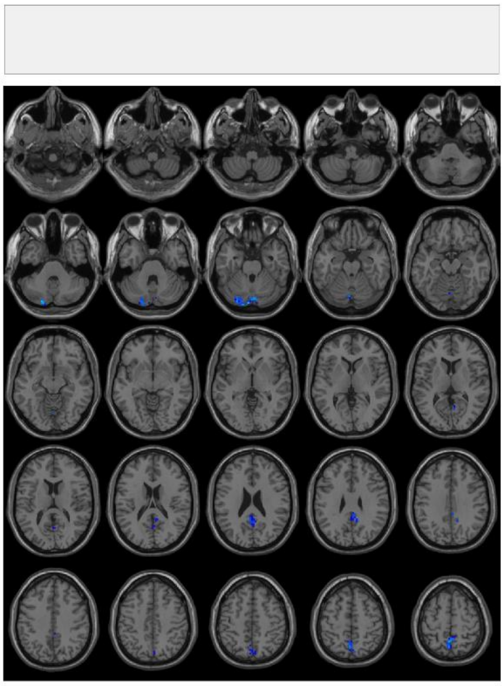

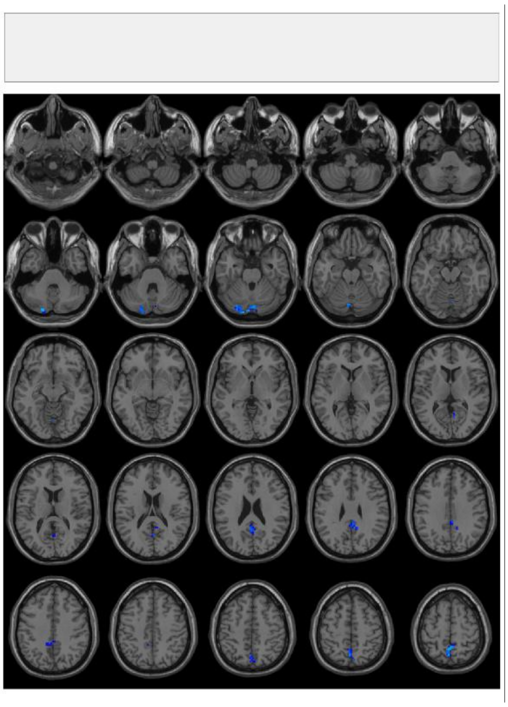

Test7

Test8

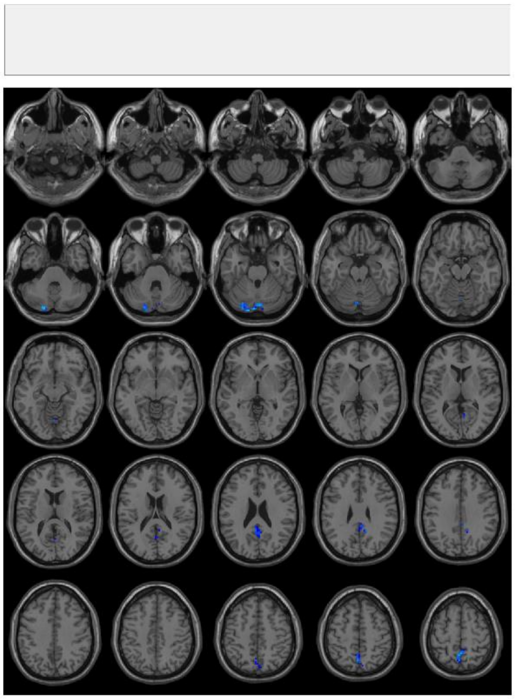

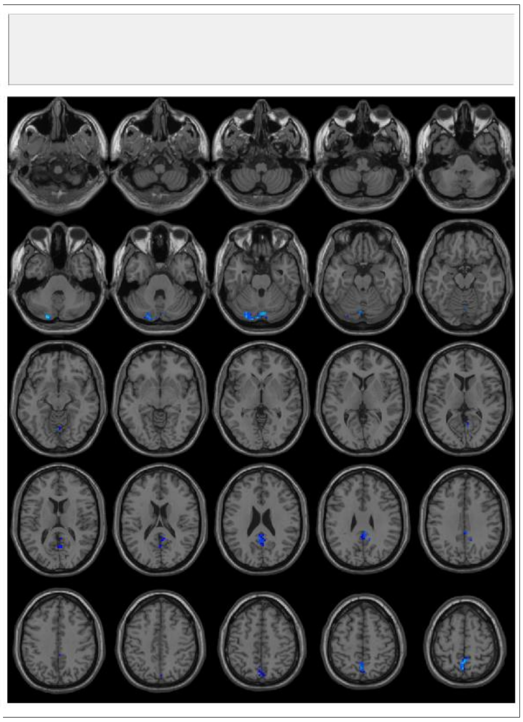

Test9

Test10
